# Seasonal-Spatial Distribution and Long-Term Variation of Transparency in Xin’anjiang Reservoir: Implications for Reservoir Management

**DOI:** 10.3390/ijerph120809492

**Published:** 2015-08-12

**Authors:** Zhixu Wu, Yunlin Zhang, Yongqiang Zhou, Mingliang Liu, Kun Shi, Zuoming Yu

**Affiliations:** 1 Chun’an Environmental Monitoring Station, Hangzhou 311700, China; E-Mail: caepb@126.com; 2 Taihu Laboratory for Lake Ecosystem Research, State Key Laboratory of Lake Science and Environment, Nanjing Institute of Geography and Limnology, Chinese Academy of Sciences, Nanjing 210008, China; E-Mails: yohnchou917251@126.com (Y.Z.); kshi@niglas.ac.cn (K.S.); 3 College of Resources and Environment, University of Chinese Academy of Sciences, Beijing 100049, China; 4 Institute of Environmental Protection Science, Hangzhou 310014, China; E-Mails: lmllyx@163.com (M.L.); yzm0571@163.com (Z.Y.)

**Keywords:** chlorophyll *a*, long-term trend, rainfall, water transparency, Xin’anjiang Reservoir

## Abstract

Water transparency is a useful indicator of water quality or productivity and is widely used to detect long-term changes in the water quality and eutrophication of lake ecosystems. Based on short-term spatial observations in the spring, summer, and winter and on long-term site-specific observation from 1988 to 2013, the spatial, seasonal, long-term variations, and the factors affecting transparency are presented for Xin’anjiang Reservoir (China). Spatially, transparency was high in the open water but low in the bays and the inflowing river mouths, reflecting the effect of river runoff. The seasonal effects were distinct, with lower values in the summer than in the winter, most likely due to river runoff and phytoplankton biomass increases. The transparency decreased significantly with a linear slope of 0.079 m/year, indicating a 2.05 m decrease and a marked decrease in water quality. A marked increase occurred in chlorophyll *a* (Chl*a*) concentration, and a significant correlation was found between the transparency and Chl*a* concentration, indicating that phytoplankton biomass can partially explain the long-term trend of transparency in Xin’anjiang Reservoir. The river input and phytoplankton biomass increase were associated with soil erosion and nutrient loss in the catchment. Our study will support future management of water quality in Xin’anjiang Reservoir.

## 1. Introduction

Water transparency is the most straightforward way to evaluate the underwater light environment in aquatic ecosystems [1,2,3]. Due to its simplicity and low cost, transparency as measured by the Secchi disk depth (SDD) has been widely used by limnologists since 1865 to assess water quality [4]. Therefore, Hutchinson has stated that “A skilful limnologist can probably learn more about the nature of a lake (river) from a series of oxygen determinations combining Secchi disc and water color than from any other type of chemical data” [4].

In summary, transparency plays an important role in the following four aspects of water quality. First, transparency is a direct and important indicator of eutrophication and water quality assessment. In 1977, Carlson proposed a trophic state indicator for lakes based on water transparency as measured by the Secchi disk [5]. At present, transparency and the total phosphorus (TP), total nitrogen (TN), chemical oxygen demand (COD), and chlorophyll *a* (Chl*a*) concentrations are widely considered indicators of eutrophication assessment [6,7,8]. Second, light availability plays a critical role in regulating the distribution of submerged aquatic vegetation (SAV) and maintaining a healthy and stable macrophyte-dominated lake ecosystem [9,10]. Therefore, many studies have indicated that the maximum colonization depth and biomass of SAV are influenced primarily by water transparency [11,12]. Third, transparency provides a highly relevant measure of the extent of the euphotic water layer where primary production is possible. If combined with bathymetric information, transparency can provide an estimate of not only the volume of the habitat of phytoplankton but also the potential extent of benthic habitat with primary productivity [13,14]. Fourth, transparency is one of the important factors affecting a lake’s thermal structure, including the onset and end time of stratification, the length of the thermal stratification duration and thermocline depth and thickness [15,16]. Thermal stratification further determines the penetration depth of dissolved oxygen and the lake sediment aerobic and anaerobic environments as linked to nutrient release [17,18]. Therefore, water transparency is considered an indirect major factor affecting internal nutrient release in deep lakes and reservoirs. In recent decades, a significant transparency decrease has been observed in many oceans, coastal and inland waters due to eutrophication and human activities [19,20,21,22].

Xin’anjiang Reservoir is a nationally protected drinking water source due to its good water quality. In the future, a diversion project from Xin’anjiang Reservoir to Hangzhou and Shanghai will be built to meet the increasing need of urban inhabitants for good drinking water. However, Xin’anjiang Reservoir also suffers from the dual pressures of eutrophication and climate changes, similar to other waters around the world [8,15,18]. Water quality problems have been documented, and short-term algal blooms have appeared in the reservoir since the 1990s and have been primarily attributed to increases in nitrogen and phosphorus loading following the conversion of land in the watershed to agricultural and urban uses [23,24]. Climate warming has exerted a great effect on reservoir thermal structure and stratification [15], which will ultimately affect dissolved oxygen stratification and water quality [17,18]. The effect of increasing nutrients and climate warming on the water quality in Xin’anjiang Reservoir will be reflected through transparency. There are some scattered reports about transparency in Xin’anjiang Reservoir [8,15,25]. However, no systematic studies have been conducted to present the variation in and factors affecting transparency. Detailed SDD characterization could provide valuable information on the physico-chemical and biological processes, contributing significantly to assess their ecological conditions with important implications in reservoir management. For example, trophic state indicator covering SDD is an important reservoir management tool [26].

Therefore, given the important roles of transparency in water quality, eutrophication assessment and reservoir management, a potential novelty of this study lied in providing some insights for reservoir water quality management through revealing the causes affecting the spatial, seasonal, and long-term variability of water transparency. The aim of this study was to (1) present the spatial distribution of water transparency over different seasons, (2) analyze the seasonal variability and long-term trends in water transparency from 1988 to 2013, and (3) discuss the factors potentially affecting transparency variation and suggest management options to improve transparency for reservoir management.

## 2. Materials and Methods

### 2.1. Study reservoir and observation sites

**Figure 1:**
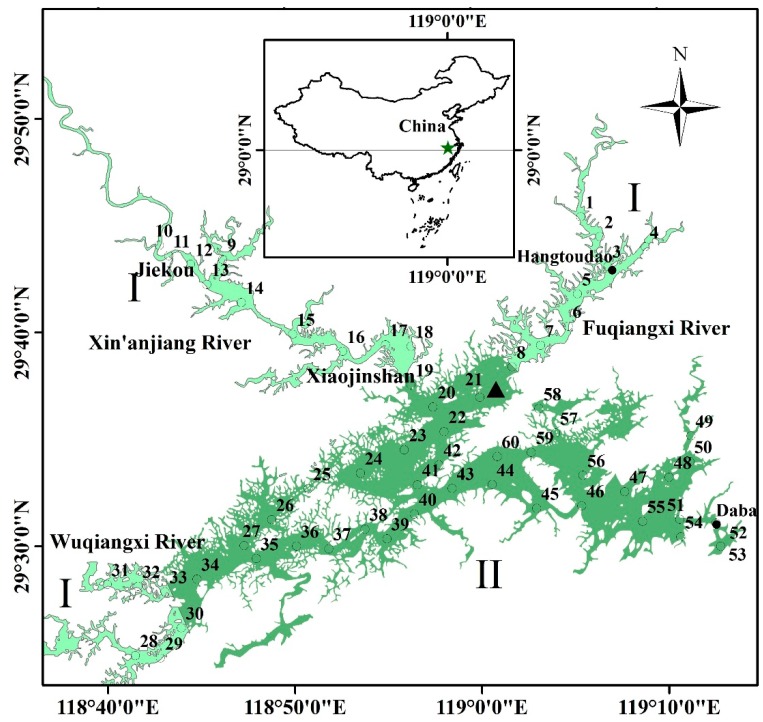
Location of Qiandaohu in China and the locations of Chun’an meteorological station (▲) and the transparency observation sites from 1988 to 2013 showing the two data sources: (●) from 1988–2013, the observation sites were located in one inlet (Hangtoudao), and the only outlet of the reservoir (Daba) was used for long-term specific observations; (○) in 2013 and 2014, the observation sites were evenly distributed around the reservoir in winter, spring and summer. The 60 sites were classified into two groups. Type I: located in the adjacent waters of the three incoming rivers, including sites 1–19 and 28–33. Type II: located in the main body of the reservoir, including sites 20–27 and 34–60.

Xin’anjiang Reservoir (29°22′–29°50′ N, 118°36′–119°14′ E) was built in 1959 and is located in western Zhejiang Province and southern Anhui Province (Figure 1, star in the upper figure). The reservoir is long and narrow, with many bays, and the greatest length and width of its bays are 150 km and 50 km, respectively. In the reservoir, there are numerous islands (another name given to this reservoir, “Qiandao”, means one thousand islands). Xin’anjiang Reservoir has a water area of 580 km^2^, a mean depth of 34 m, a water volume of 178.4 × 108 m^3^, and a catchment area of 10,480 km^2^ when the water level is 108 m. Xin’anjiang Reservoir provides many ecosystem services and economic development functions including drinking water supply, flood alleviation, power production, and shipping. In addition, the reservoir is an important recreational and aesthetic resource that adds to the economic vitality and quality of life for Chun’an County. Therefore, the protection and long-term monitoring of reservoir water quality are major concerns for local agencies and citizen groups.

### 2.2. Short-Term Observations of Spatial Distribution of Transparency

To describe the subtle features of the spatial distribution of water transparency, we conducted three cruise investigations of transparency and Chl*a* during three different seasons (spring, summer and winter) in Xin’anjiang Reservoir. Sixty sites (○) were defined, and surface water samples (0.5 m) were collected around the reservoir from 29 November to 1 December in 2013 (winter), from 27 to 29 May (spring) and from 31 July to 2 August (summer) in 2014 and 2015 (Figure 1). Because natural and climatological backgrounds, including temperature, rainfall, and vegetation cover, in autumn are similar to those in spring in temperate and subtropical regions, autumn was not considered in this study. To address spatial differences in transparency and the effect of the inflowing river on transparency, the 60 sites were classified as follows: Type I, located in the waters adjacent to the three incoming rivers, including sites 1–19 and 28–33; and Type II, located at the main body of the reservoir, including sites 20–27 and 34–60 (Figure 1). In addition, the specific sites 1–8, 10–19, 28–33, 20–45 and 60, 46–59 were located in the northeast zone, northwest zone, southwest zone, central zone, and eastern zone, respectively.

### 2.3. Long-Term Site-Specific Observations

A 26-year study, from 1988 to 2013, was conducted at Daba (the outlet of the reservoir) and Hangtoudao (one inlet of the reservoir) during the first 10-day period of each month in Xin’anjiang Reservoir (Figure 1). At Daba, research cruises were conducted in April, July and December, representing spring, summer and winter, respectively. At Hangtoudao, the research cruises were conducted only in July (summer). In addition, the transparency was measured from 1988 to 2013, but the TN and Chl*a* concentrations were measured from 2001 to 2013.

To discuss the effect of rainfall and temperature on the seasonal variation and long-term trend of transparency, we downloaded the monthly rainfall and air temperature data from the China meteorological data-sharing service system. We obtained data from Chun’an meteorological station (29°37′ N, 119°01′ E) (▲ in Figure 1), which is located at Xin’anjiang Reservoir and has daily, monthly and yearly meteorological data. The monthly average data were considered on a seasonal basis, with the seasons defined as follows: winter, December–February; spring, March–May; and summer, June–August.

### 2.4. Measurement of Transparency, TN and Chla

The observation sites were positioned using a Global Positioning System (GPS) with an accuracy of 2.0 m. A standard 30-cm diameter Secchi disk was used to measure SDD [4]. Water samples for Chl*a* (1000–5000 ml, according to the amount of phytoplankton) were filtered through Whatman GF/F fiberglass filters. Chl*a* was extracted with ethanol (90%) at 80 ºC and analyzed spectrophotometrically at 750 and 665 nm with correction for phaeophytin-*a* [27]. The TN was determined by spectrophotometry after digestion with alkaline potassium persulfate according to the procedures for “Standard Methods for the Examination of Water and Wastewater” [28].

### 2.5. Data Analysis

Statistical analyses, including mean values, linear and non-linear fitting, and regression and correlation analyses, were performed using the Statistical Program for Social Sciences (SPSS) 17.0 software. Because the numbers of Type I and Type II sites and the long-term and short-term monitoring differed substantially, the differences in the parameters between Type I and Type II and between long-term and short-term monitoring were assessed with independent sample *t*-tests. A one-way analysis of variance (ANOVA) was used to compare the seasonal differences in parameters with the similar or same sampling number. The significance levels are reported as significant if *p* < 0.05. The slope of the linear regression fitting of transparency *vs*. year gave the rate of change of transparency in Xin’anjiang Reservoir.

Contour maps showing the spatial distribution of transparency were generated using inverse distance weighing interpolation technique in ArcGIS 9.2 (ESRI, Redlands, California, CA, USA).

## 3. Results

### 3.1. Spatial Distribution

**Figure 2:**
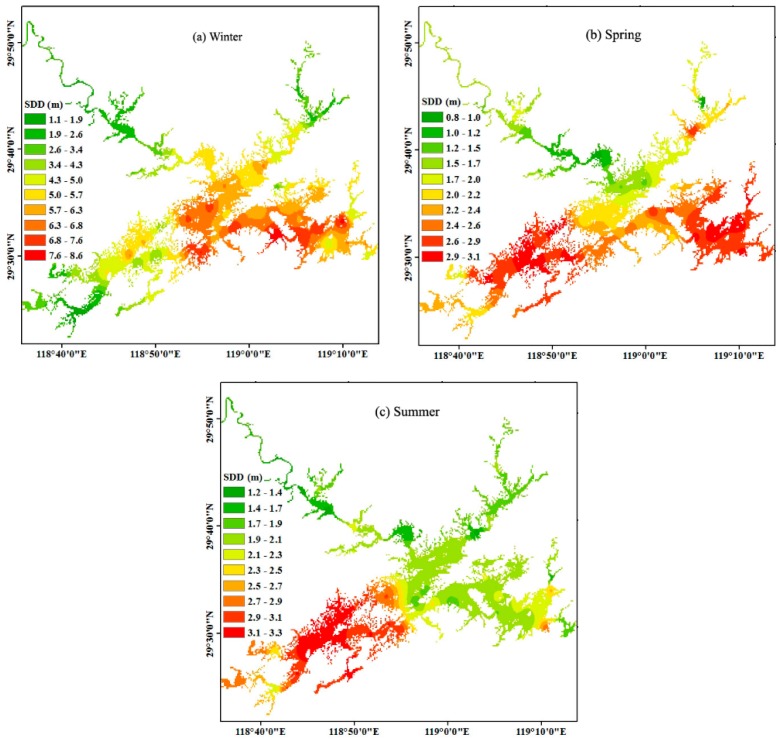
Spatial distribution of transparency in the winter of 2013 (**a**), spring of 2014 (**b**), and summer of 2014 (**c**) in Xin’anjiang Reservoir.

**Figure 3:**
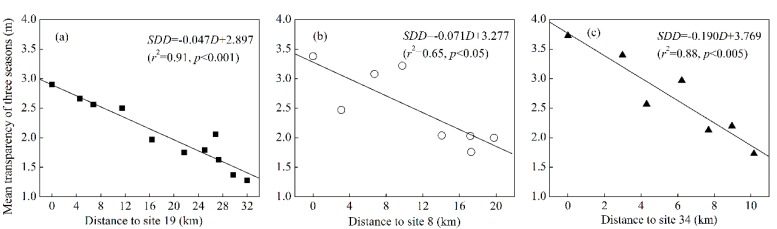
Linear relationships between the mean transparency of three seasons from 2013 to 2014 and the distance to site 19 for the Xin’anjiang River (sites 9–19) (**a**), to site 8 for the Fuqiangxi River (sites 1–8) (**b**), and to site 34 for the Wuqiangxi River (sites 28–34) (**c**).

Figure 2a–c shows the substantial spatial differences among transparency distributions in the spring, summer, and winter with two different types are considered. The first type (I) of low transparency is characteristic of river mouths, including Xin’anjiang River, Wuqiangxi River and Fuqiangxi River, which represent 60%, 20% and 10%, respectively, of the annual mean incoming runoff of Xin’anjiang Reservoir (Figure 1 and Figure 2a–c). The river mouths are distinguished by low values of transparency for most of the year, less than 3 m in most cases. The average transparency of the first type was 2.30 ± 1.21 m. From the upstream reaches of the three incoming rivers to the open water around the reservoir, transparency gradually increases. For example, the water transparency is greatly affected by the Xin’anjiang River and maintains a very low value from the upstream site 10 at Jiekou (1.28 ± 0.10 m) to a high value in the open site 19 at Xiaojinshan (2.90 ± 2.27 m) along more than 30 km for all three seasons. To qualitatively assess the effect of the inflowing river on the distribution of transparency, we performed a linear regression between transparency and the distance to the open water. In detail, the distances to site 19 for the Xin’anjiang River, to site 8 for the Fuqiangxi River, and to site 34 for the Wuqiangxi River were calculated. A significant linear relationship was found between the transparency and the distance to the open water for the Xin’anjiang River for sites 9–19 (*r*^2^ = 0.97, *p* < 0.001), the Fuqiangxi River for sites 1–8 (*r*^2^ = 0.65, *p* < 0.05), and the Wuqiangxi River for sites 28-34 (*r*^2^ = 0.88, *p* < 0.005) (Figure 3), indicating the significant effect of the incoming river on the transparency in Xin’anjiang Reservoir.

The second type (II) of high transparency was characteristic of the central and eastern discharge zones of Xin’anjiang Reservoir (Figure 2a–c), with an average value of 3.62 ± 1.89 m, significantly higher than that of the first type (2.30 ± 1.21 m) (t-test, *p* < 0.001). This zone is the deep area and is far from the sources of suspended matter of the incoming river. In this zone, the water transparency is slightly affected by the incoming river runoff due to sedimentation during long-distance transport. In contrast, other factors, such as phytoplankton growth, stratification and mixing, will contribute more to transparency. This area featured the largest transparency, as high as 8.6 m in winter. For the second type, the spatial distribution exhibited some differences in the three seasons. In summer, the highest transparency was distributed in the central zone (sites 20–45 and 60) and not in the eastern zone (sites 46–59) of Xin’anjiang Reservoir (Figure 2c). In contrast, the highest transparency was distributed in the eastern zone in spring and winter.

### 3.2. Seasonal Variation

**Figure 4:**
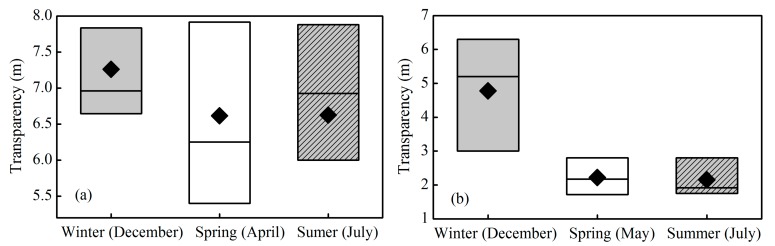
Boxplots of transparency at Daba in the spring, summer and winter in Xin’anjiang Reservoir for long-term observations over the 26-year period (1988 to 2013) (**a**) and short-term observations from 2013 to 2014 (**b**). The box is determined by the 25th and 75th percentiles, and the values of the median (horizontal line) and mean (diamond) are also included.

The seasonal variations of water transparency at Daba over the 26-year period from 1988 to 2013 and for 60 sites from 2013 to 2014 in Xin’anjiang Reservoir are shown as boxplots in Figure 4. The inter-annual average values of transparency were 7.26 ± 1.22, 6.61 ± 1.70, and 6.62 ± 1.48 m in winter, spring, and summer, respectively (Figure 4a). There was almost no difference in the average values of transparency in spring and summer. However, the average value of transparency was markedly higher in winter than that in spring and summer, although these differences were not statistically significant (one-way ANOVA, *p* ˃ 0.05).

Similar to the long-term specific-site observations, transparency exhibited a seasonal difference based on the three short-term observations. The average transparency was 4.78 ± 1.99 m in winter, significantly higher than 2.22 ± 0.64 m and 2.16 ± 0.59 m in spring and summer, respectively (Figure 4b) (one-way ANOVA, *p* < 0.001). However, there was no statistically significant difference between spring and summer (one-way ANOVA, *p* = 0.562). The seasonal pattern based on three short-term observations at 60 sites was similar to that based on the long-term specific site observations at Daba from 1988 to 2013.

**Figure 5:**
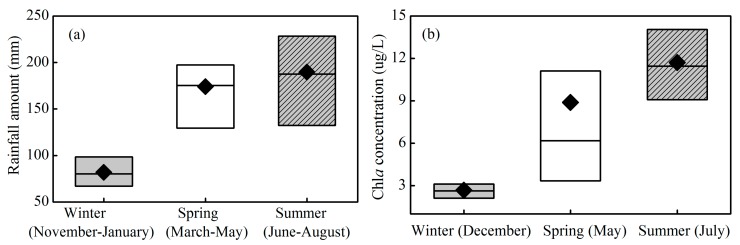
Boxplots of the monthly average rainfall in Xin’anjiang Reservoir for the long-term observations over the 26-year period (1988 to 2013) (**a**) and the chlorophyll *a* concentration (**b**) for the short-term observations from 2013 to 2014. The box is determined by the 25th and 75th percentiles, and the values of the median (horizontal line) and mean (diamond) are also included.

To analyze the reasons for the seasonal variation in transparency, Figure 5 presents the seasonal characteristics of the monthly average rainfall amount over the 26-year period from 1988 to 2013 and the Chl*a* concentration at 60 sites from 2013 to 2014 in Xin’anjiang Reservoir. The catchment monthly average rainfall amount was 81.9 ± 24.3 mm in winter, significantly lower than 174.1 ± 53.5 mm and 189.6 ± 75.5 mm in spring and summer, respectively, from 1988 to 2013 (Figure 5a) (one-way ANOVA, *p* < 0.001). The short-term spatial distribution observations at 60 sites showed that the Chl*a* concentration was 2.67 ± 0.89 µg/L in the winter of 2013, significantly lower than the values of 8.88 ± 7.55 and 11.71 ± 3.86 µg/L in spring and summer, respectively, in 2014 (Figure 5b) (one-way ANOVA, *p* < 0.001).

### 3.3. Long-Term Trends

**Figure 6:**
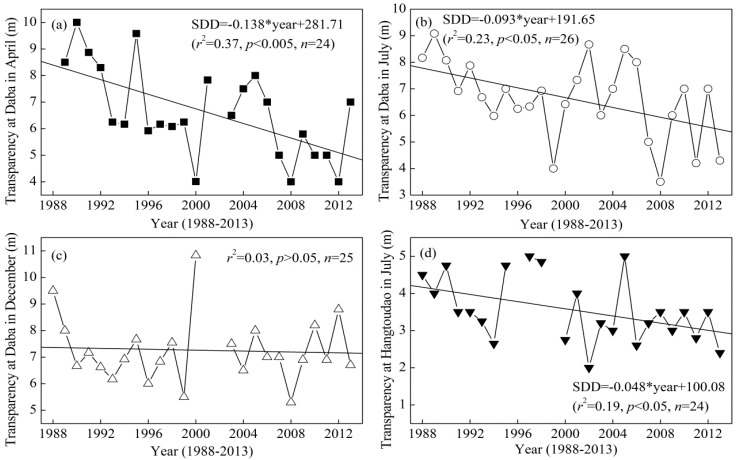
Long-term trends of transparency at Daba in April (**a**), July (**b**), and December (**c**) and at Hangtoudao in July (**d**) in Xin’anjiang Reservoir from 1988 to 2013.

The long-term trends of transparency in the last 26 years (1988–2013) at Daba in April, July, and December and at Hangtoudao in July in Xin’anjiang Reservoir are shown in Figure 6. The long-term monitoring data for transparency showed that significant changes had occurred in the underwater light environment of Xin’anjiang Reservoir. During these 26 years, a significant decrease (*p* < 0.05) in transparency occurred at Daba in April and July (Figure 6a,b) and at Hangtoudao in July (Figure 6d). However, no significant decrease was found at Daba in December (Figure 6c). The decreased rate, as shown by the slope of the line, varied considerably among the different sites and seasons, with the greatest decrease in transparency at 1.38 m per decade, which was based on the linear fitting as recorded at Daba in April (Figure 6a). When all three seasons were considered together at Daba, the mean decrease in transparency was 0.79 m per decade, which was statistically significant (*r*^2^ = 0.28, *p* ≤ 0.005), indicating a 2.05 m decrease in water transparency in the past 26 years.

**Figure 7:**
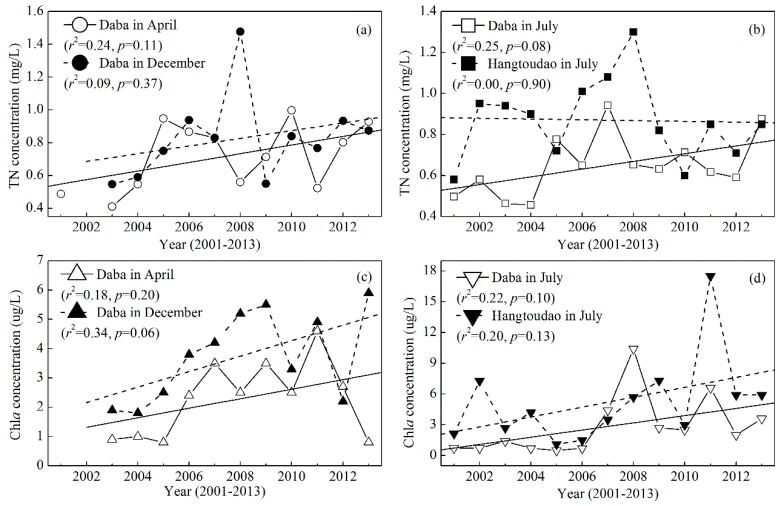
Long-term trends for the total nitrogen (**a**), (**b**) and chlorophyll *a* (**c**), (**d**) concentrations at Daba in April, July, and December and at Hangtoudao in July in Xin’anjiang Reservoir from 2001 to 2013.

To discuss whether reservoir eutrophication and phytoplankton biomass could explain the long-term variation in transparency, we presented the long-term trends of the TN and Chl*a* concentrations from 2001 to 2013 (Figure 7). The TN concentration markedly increased at Daba in April, July and December (Figure 7a,b) but had almost no change at Hangtoudao in July (Figure 7b). Meanwhile, the Chl*a* concentration markedly increased at Daba in April, July and December and at Hangtoudao in July (Figure 7c,d). However, none of these linearly increasing trends were statistically significant (*p* > 0.05) (Figure 7). The long-term trends of the TN and Chl*a* concentrations contrasted with those of transparency, which partially explains the decrease in transparency.

## 4. Discussion

### 4.1. Spatial and Seasonal Variations

Water transparency depends on the incoming river runoff near the river mouths, the life cycles of the planktonic communities, the resuspension of the silty bottom sediments, and the local depth, as well as stratification and mixing [29,30,31]. Spatially, the lower transparency around river mouths than that of the central and eastern discharge zones of Xin’anjiang Reservoir and the significant linear relationship between transparency and the distance to the open water confirmed the effect of the incoming river runoff on transparency. The seasonal variation in transparency in Xin’anjiang Reservoir was consistent with the previous results from other reservoirs and lakes [32,33]. For example, the greatest transparency occurred in February and March, whereas the lowest occurred in July and August in Lake Mead, the largest reservoir in the United States [32], demonstrating the effect of phytoplankton biomass on transparency.

The seasonal variations in transparency in Xin’anjiang Reservoir may be attributed to two causes. First, the transparency was greatly affected by the runoff from the catchment, which carried a large amount of suspended matter. The significantly low rainfall in the winter decreased the inflowing runoff, which caused a decrease in the total suspended matter (TSM) and an increase in the transparency. A similar study has found that the transparency increased with the decreased rainfall [34,35]. Second, the phytoplankton biomass decreased in the winter due to water temperature and light limitation [14,36], which would increase the transparency. The short-term spatial distribution observations for 60 sites showed that the Chl*a* concentration was 2.67 ± 0.89, 8.88 ± 7.55, and 11.71 ± 3.86 µg/L in the winter of 2013 and in the spring and summer of 2014, respectively, which corresponded well with the contrasting trend in transparency of 4.78 ± 1.97, 2.22 ± 0.64, and 2.16 ± 0.59 m. Similarly, LaBounty [32] found that transparency in the open water of Las Vegas Bay ranged from <1 m during the growing season to 5–7 m in winter due to the effect of phytoplankton biomass based on transparency data between July 1990 and December 2007.

### 4.2. Factors Affecting the Long-Term Trend

A decrease of 2.05 m in the water transparency in the past 26 years in Xin’anjiang Reservoir further confirmed the decreasing trend of transparency in many inland waters around the world in recent decades due to environmental change and human activities [19,20,22].

**Figure 8:**
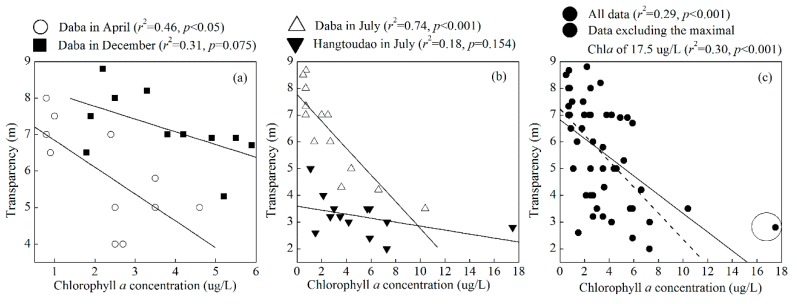
Correlations between the chlorophyll *a* concentration and the transparency from 2001 to 2013 in Xin’anjiang Reservoir. The dotted line shows the linear fitting excluding the maximal chlorophyll *a* of 17.5 µg/L as labeled in the cycle.

To further analyze the effect of the phytoplankton biomass on transparency, we performed a correlation analysis and found negative linear relationships between the Chl*a* concentration and transparency (Figure 8). For Daba in April and July, and when all of the data including those from Daba and Hangtoudao were considered together, the linear relationships were statistically significant (*p* < 0.05). Considering that the maximal Chl*a* of 17.5 µg/L is significantly higher than the second highest Chl*a* of 10.4 µg/L and that the median value is 2.7 µg/L, we fit Chl*a* and transparency, excluding the maximal Chl*a* of 17.5 µg/L. The linear relationship was still statistically significant (*p* < 0.05) (Figure 8c). In addition, the low air temperature inhibiting phytoplankton growth can partially explain the anomalous high value in 2000 (Figure 8c). Considering that the transparency investigation was carried out in the first ten-day period of a month, we found the monthly average air temperature for November (a month advance of transparency investigation) in 2000 was the second lowest value during the period of 1988–2013. Therefore, these results further demonstrate that a phytoplankton biomass increase could partially explain the decreased transparency in the past 26 years. Many previous studies have also reported that a transparency decrease was linked to an increase in the phytoplankton biomass in ocean, coastal and inland waters [29,32,37,38]. For example, water transparency in the open Baltic Sea has decreased during the last one hundred years, and 13–17% variability in transparency was caused by phytoplankton [29]. In addition, the increased TN and Chl*a* concentrations and the decreased transparency jointly confirm that Xin’anjiang Reservoir underwent accelerating eutrophication.

The relatively low degree of determination between Chl*a* and transparency indicates that TSM substantially contributed to transparency in Xin’anjiang Reservoir. Although we have no synchronous TSM data that reject the explanation that TSM contributed to transparency, the aforementioned significant negative correlations between transparency and distance to open water demonstrate the important contribution of TSM from river catchments in regulating transparency. In addition, some studies have shown that the water transparency was affected by TSM, which had been linked to regional rainfall of the catchment [34,35]. Therefore, we correlated the transparency at the Daba site in April, July and December to the previous one month’s rainfall. In summer, the transparency was significantly negatively correlated with the previous one month’s rainfall (*r*^2^ = 0.39, *p* < 0.01), indicating that the runoff from the catchment had a significant effect on the reservoir summer transparency. In spring, the transparency was negatively correlated with the previous one month’s rainfall but was not statistically significant (*r*^2^ = 0.07, *p* = 0.35). In contrast, there were no negative correlations between transparency and the previous one month’s rainfall in the winter. Therefore, the river input that was caused by high rainfall and the increased phytoplankton biomass in the summer jointly contributed to the long-term trend and seasonal variation in transparency in Xin’anjiang Reservoir.

### 4.3. Implications for Reservoir Management

Human-induced eutrophication is occurring throughout the world and has become the primary water quality issue for most freshwater and coastal marine ecosystems [39]. One of the primary management issues regarding inland lakes and reservoirs for local agencies is the trophic state, and of the four most common indicators of trophic state (transparency, TP, TN, and Chl*a*), transparency is very easily observed and has long-term data available from observations. Therefore, the study of transparency has potential management implications for local agencies. The decreased transparency directly or indirectly due to eutrophication and human activities indicates that the deterioration of water quality would be considered undesirable. In contrast, increased transparency would be considered desirable.

Overall, the spatial distribution and long-term trend were linked to the inflowing river input and nutrient increase from the surrounding catchment. However, in the catchment of Xin’anjiang Reservoir, the increase in vegetation in the headwaters is far from complete. The land use change from natural forest land to tea gardens has decreased the vegetation cover and increased the potential for soil erosion and nutrient loss [40]. Therefore, water and soil conservation in the catchment to reduce soil erosion and nutrient loss is a prerequisite for the improvement of SDD in the Xin’anjiang Reservoir [23,24]. In addition, tourism development and urbanization in Chun’an county and upstream in Huangshan city caused increased nutrient inputs and a change in land use from vegetation to construction land [40,41]. Therefore, it is urgent to make measurements in support of measures that will inhibit development around the reservoir and in the sensitive regions of soil erosion and nutrient loss. It is necessary to constructed wetlands to intercept nutrient and soil loss in the river floodplain. In summary, some reservoir management measures should be implemented to improve transparency and protect water quality: (i) protecting the vegetation and controlling soil erosion in the catchment; (ii) reducing the external nutrient inputs from agricultural activities, industrial waste, and domestic wastewater; (iii) preventing the development of construction land on the reservoir shores; and (iv) promoting vegetation growth on the reservoir shoreline. Therefore, the potential novelty and contribution of this study are to provide a systematic analysis of the spatial distribution, seasonal variation and long-term trend, elucidating the affecting factors of transparency, and proposing some detailed management strategies to improve transparency in Xin’anjiang Reservoir.

Future investigators and managers will be responsible for sorting out the various anthropogenic and nonanthropogenic factors to determine the reason for any change in transparency. Additionally, the physical and chemical limnology of Xin’anjiang Reservoir should be more extensively investigated and modeled mathematically to provide scientific support for water quality management and protection.

## 5. Conclusions

Transparency has been widely used to provide an integrative indicator describing a combination of eutrophication-related characteristics, together with indicators of suspended matter, phytoplankton biomass and primary production. For the effective water quality management of Xin’anjiang Reservoir, it is essential to have accurate information on the temporal-spatial distribution and long-term trends of transparency. Spatially, water transparency increases with the distance from the shore and riverine mouth. Seasonally, the lowest transparency is recorded in the summer and is accompanied by increased river runoff and phytoplankton biomass in most cases. Our results further demonstrate that a significant decrease in transparency occurred in Xin’anjiang Reservoir over the past 26 years due to the accelerating eutrophication and watershed inputs. Therefore, some management strategies, including soil conservation, agricultural activities and livestock-raising regulations, should be strengthened by the local government to decrease the input of nutrients and TSM from the catchment. Our study will make a great contribution to reservoir water quality management for the local government authorities by elucidating the potential causes affecting the seasonal, spatial and long-term variations of transparency, and proposing some management strategies.
